# Supplementary material for: The increasing importance of fellowships and career development awards in the careers of early-stage biomedical academic researchers
Source: PLoS One. 2019 Oct 17;14(10):e0223876. doi: 10.1371/journal.pone.0223876 (PMC6797166; doi:10.1371/journal.pone.0223876)
Supplement: Attachment — Submitted filename: Response to reviewers 2.docx [file pone.0223876.s010.docx]

**Reviewer 1**

- The author has addressed previous critiques well and only one minor issue remains for this reviewer. The author indicates that "F32 awardees are nearly 6 percent more likely to receive an R01 than those who tried and failed to receive an F32...." as well as similar statements for other award mechanisms. However, the data available in NIH ExPORTER are only those applications that are funded and there is no indication in these data of unsuccessful applications for grant awards. Therefore, it is not obvious how this conclusion is reached. If others have performed such a study, a citation must be provided. Otherwise, just because a first-time R01 awardee did not have a previous F32 (or other training award) does not mean that he or she tried and failed as there are other mechanisms of support and perhaps the awardee never even applied for an F32 (or other training award).

The data quoted in the indicated sentence are from previously published papers—Heggeness et al and Nikaj and Lund, references 12 and 14 respectively. I forgot to include these references in my revision, and I thank the reviewer for pointing this out. I have rectified this issue.
